# Antimicrobial Peptides Targeting Gram-Positive Bacteria

**DOI:** 10.3390/ph9030059

**Published:** 2016-09-20

**Authors:** Nermina Malanovic, Karl Lohner

**Affiliations:** 1 Institute of Molecular Biosciences, Biophysics Division, University of Graz, NAWI Graz, Humboldtstrasse 50/III, 8010 Graz, Austria; 2 BioTechMed Graz, Humboldtstrasse 50/III, 8010 Graz, Austria

**Keywords:** cell wall, lipoteichoic acid, peptidoglycan biosynthesis, membrane phospholipids, mode of action of AMPs

## Abstract

Antimicrobial peptides (AMPs) have remarkably different structures as well as biological activity profiles, whereupon most of these peptides are supposed to kill bacteria via membrane damage. In order to understand their molecular mechanism and target cell specificity for Gram-positive bacteria, it is essential to consider the architecture of their cell envelopes. Before AMPs can interact with the cytoplasmic membrane of Gram-positive bacteria, they have to traverse the cell wall composed of wall- and lipoteichoic acids and peptidoglycan. While interaction of AMPs with peptidoglycan might rather facilitate penetration, interaction with anionic teichoic acids may act as either a trap for AMPs or a ladder for a route to the cytoplasmic membrane. Interaction with the cytoplasmic membrane frequently leads to lipid segregation affecting membrane domain organization, which affects membrane permeability, inhibits cell division processes or leads to delocalization of essential peripheral membrane proteins. Further, precursors of cell wall components, especially the highly conserved lipid II, are directly targeted by AMPs. Thereby, the peptides do not inhibit peptidoglycan synthesis via binding to proteins like common antibiotics, but form a complex with the precursor molecule, which in addition can promote pore formation and membrane disruption. Thus, the multifaceted mode of actions will make AMPs superior to antibiotics that act only on one specific target.

## 1. Introduction

For many years, the World Health Organization has emphasized the high proportions of antibiotic resistance in bacteria causing common infections such as pneumonia, urinary tract and blood stream infections in all regions of the world. Furthermore, their global report on surveillance in 2014 referred to alarming levels in bacterial resistance in many parts of the world and the urgent need to develop a global intervention plan against antimicrobial resistance [1]. One of the problematic microbes that causes a high percentage of hospital-acquired infections is the Gram-positive strain *Staphylococcus aureus*, which is resistant to a number of antibiotics, in particular methicillin. Although in general less Gram-positive bacteria are pathogenic to humans, some like *Streptoccocus* and *Staphylococcus*, which normally reside on healthy skin or in the nasopharyngeal region, can become pathogenic at certain conditions initiating skin infections, followed by sepsis, pneumonia, meningitis and in the worst case death [2]. The same is true for gut residing *Enterococcus* and *Clostridia* species. The former are associated with urinal infection in patients with catheter or catheter-related bloodstream infections and the latter is causing severe diarrhea by prolonged treatment with antibiotics or proton pump inhibitors [3,4]. In fact, a study involving 19 US hospitals during 2007 and 2010 revealed that the percentage of isolates of *E. faecium* being resistant to vancomycin was highest (87.1%) followed by *S. aureus* resistance to oxacillin–methicillin (56.8%) [5]. Pathogenicity of the foodborne *Listeria monocytogenes* is described to cause listeriosis, which mainly affects high-risk individuals such as pregnant women affected by the case of invasive infection [6]. Moreover, cases of pathogenic relevance of *Lactobacillus* strains, which are considered to be harmless, are also known [7]. Finally, tuberculosis is caused by the Gram-variable acid-fast *Mycobacterium tuberculosis,* which not only infects the lung, but also many other organs and tissues. Although treatment with conventional antibiotics is successful, tuberculosis is still one of the bacterial infections with the worldwide highest disease burden killing 1.5 million people per year [8,9]. The potential of antimicrobial peptides (AMPs) as novel therapeutic strategies in the treatment of tuberculosis was discussed in a recent review [10]. To conclude, in general, any bacterial strain may become pathogenic.

Thus, with the threat of a post-antibiotic era, research on host defense (antimicrobial) peptides [11,12,13,14,15] has markedly increased, as reflected by the more than 2600 natural or synthetic peptides with antimicrobial activity listed in the Antimicrobial Peptide Database (APD2, http://aps.unmc.edu/AP) [16]. Within the last decades, the number of new antibiotics steadily decreased and only recently two novel antibiotics, the antimicrobial lipopeptides daptomycin and oritavancin, were approved by the FDA for the treatment of severe infections caused by Gram-positive bacteria. The efficacy of daptomycin, produced by the soil bacterium *Streptomyces roseosporus*, against methicillin-resistant *S. aureus* was recently summarized [17]. Albeit oritavancin, a semisynthetic lipoglycopeptide, was only approved by FDA for the treatment of acute bacterial skin and skin structure infections, it is also a potential candidate where new innovative therapy is needed such as infective endocarditis and osteomyelitis [18]. The advantage in developing AMPs as therapeutic agents is that as such peptides are an intrinsic part of most living organisms [19,20,21], providing a first line of defense against a substantial array of pathogenic microorganisms, by direct killing as well as modulation of the immune response [22,23,24,25]. Owing to the fact that these molecules exhibit multifaceted mechanisms of action and frequently kill bacteria within minutes, development of resistance is less likely [12,26].

A large number of AMPs are considered to kill bacteria via a membrane damaging mechanism, although intracellular targets have been also reported. In any case, independent of their mode of action, AMPs encounter several potential interaction sites on their path to their target site. Adsorption of AMPs on the bacterial surface will be mainly driven electrostatically between the cationic peptides and negatively charged cell wall components such as wall- and lipoteichoic acids of Gram-positive or lipopolysaccharides of Gram-negative bacteria. Recent studies suggested that interaction of AMPs with bacterial cell wall constituents, including peptidoglycan, may reduce the local concentrations of AMPs at the cytoplasmic membrane and in turn their efficacy [27]. In fact, enormously high peptide coverage was needed to eradicate bacteria via a membrane damaging mode as shown for *E. coli* killing by PAMP-23, a cationic amphipathic helical peptide from the cathelicidin family [28].

Clearly, lipids are involved in all these steps and their molecular properties will have a strong impact on the consequent processes [29,30]. Therefore, it is also important to consider membrane architecture and lipid composition in order to understand the molecular mechanism and target cell specificity of AMPs as well as to design proper model systems. So far, it is well accepted that the different physicochemical properties of the (phospho)lipids found in biological membranes allow AMPs to discriminate between bacterial and host cell membranes [11,15,31,32]. Cholesterol, a major component of eukaryotic but not of bacterial cell membranes, seems to also play an important role in this process. The presence of this neutral lipid in model membranes inhibited the fragmentation of lipid vesicles by cationic, linear magainin derived AMPs [33,34]. In addition, high resolution structural studies provided structures on AMPs bound to lipid micelles as reported for human cathelicidin LL-37 [35,36] or magainin peptides [37,38] to name a few. Structural NMR studies also provided experimental evidence for the mechanism of membrane disruption of these peptides [39,40,41,42,43]. In this review, the focus is on AMPs specifically acting against Gram-positive bacteria and hence emphasis is not only on the interaction of AMPs with the cytoplasmic membrane, but also with the cell wall components peptidoglycan and teichoic acids, as well as interference of AMPs with their biosynthesis. Moreover, as numerous reviews exist on membrane damaging activities of AMPs (e.g., [44] and references therein), we will not discuss in depth those mechanistic backgrounds but rather delineate mode of actions that may be relevant for Gram-positive bacteria with an emphasis on the diverse nature of bacterial cell envelopes of Gram-positive and Gram-negative bacteria.

## 2. Structural Characteristics of AMPs

The main characteristics of AMPs for high binding and selectivity toward microbial membranes have been extensively reviewed [45]. It was suggested that the amino acid composition determining the physicochemical properties of the peptide in respect to charge, amphipathicity, hydrophobicity, flexibility and H-bonding capacity are key factors for their mode of action and selectivity toward microbial cells [46]. Upon contact with microbial membranes, AMPs often undergo structural changes adopting defined secondary structures or oligomerize into aggregates that also account for the considerable diversity of antimicrobial mode of action [45]. At the end, amphipathicity, resulting from segregation of apolar and polar residues upon secondary structure formation favors internalization of the peptide and in turn membrane perturbation. Thereby, the presence of hydrophobic amino acids promotes stronger partitioning into membranes. Nevertheless, there is consensus that the positive charge of the peptide is essential for initial binding to the negatively charged bacterial membrane surface, which allows discrimination between bacterial and host cell membrane, and hydrophobicity is needed for insertion into and perturbation of the membrane [30,47].

**Figure 1:**
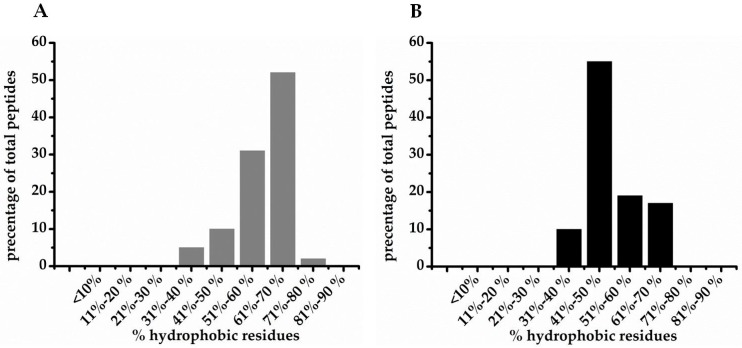
Percentage of hydrophobic residues of frog antimicrobial peptides (AMPs) in relation to the number of total peptides acting only against Gram-positive (**A**) and Gram-negative; (**B**) bacteria, respectively. The Antimicrobial Peptide Database [16] displays 401 peptides from diverse genera of frog active against both, Gram-positive and Gram-negative bacteria. Sixty one AMPs are found to be specific for Gram-positive and 42 for Gram-negative bacteria, whereby only peptides are considered with MIC < 100 μM or 100 μg/mL.

However, what are the parameters that permit discrimination between Gram-negative and Gram-positive bacteria? Searching the antimicrobial peptide database [16], no obvious preference for targeting only Gram-positive or Gram-negative bacteria in respect of hydrophobicity, length and charge of amino acid residues as well as secondary structure of AMPs could be identified. Conjointly, the vast majority of these peptides are composed of 10–50 amino acids and exhibit a positive net charge (Table 1). Approximately 13% of the entries account for larger peptides/proteins between 50 and 150 amino acid residues. For more than 60% of AMPs, the structure is not known. Further, the fraction of hydrophobic residues is mostly between 30% and 50%, although it seems that Gram-positive bacteria have a somewhat wider distribution toward higher content of hydrophobic residues. However, one has to consider that the number of AMPs being specific for Gram-positive bacteria is much larger than for Gram-negative bacteria. In addition, this summary contains peptides from various sources. Therefore, we compared the fraction of hydrophobic residues of AMPs from diverse genera of frog, which had the largest entries in the Antimicrobial Peptide Database [16]. Displaying these data supports the above assumption (Figure 1). In this context, it is of interest that it has been suggested that high hydrophobicity may prevent peptide translocation through the outer membrane of Gram-negative bacteria [48].

## 3. Architecture of Gram-Positive Cell Envelopes

**Figure 2:**
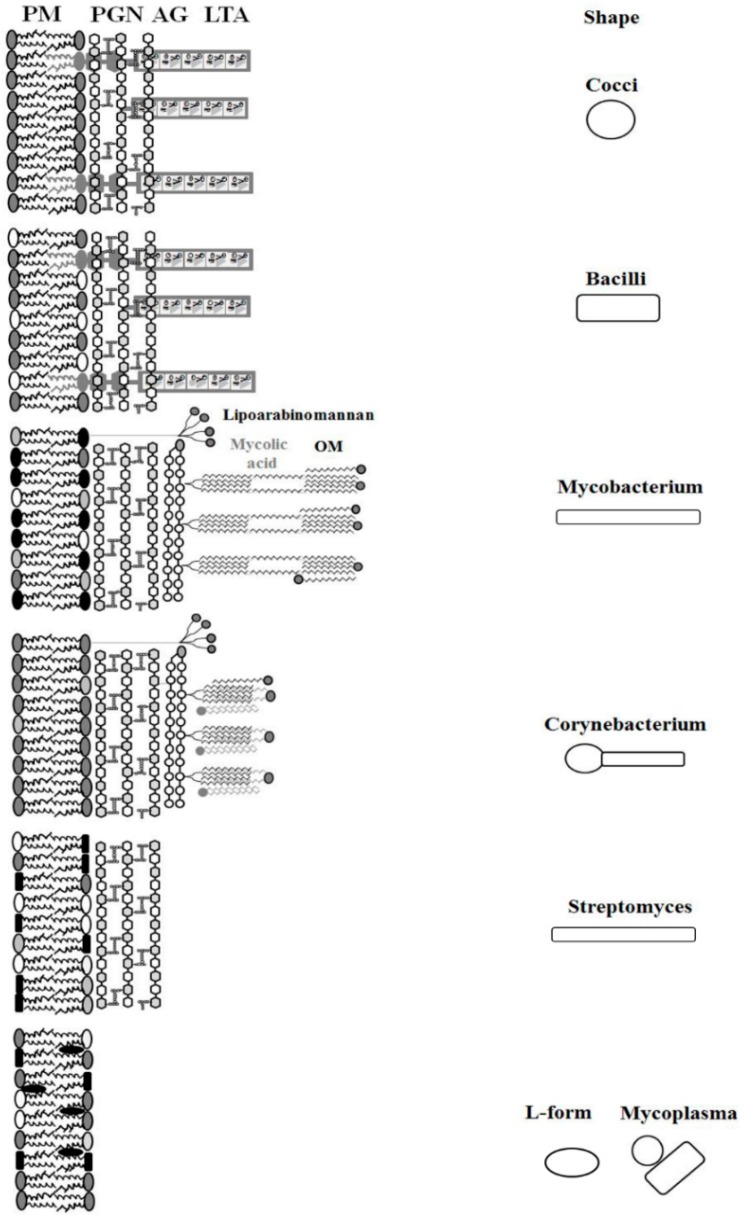
Schematic representation of membrane organization and morphology of selected Gram-positive bacteria. Gram-positive bacteria are characterized by a phospholipid bilayer of different composition (white ellipsoid, neutral phospholipid; grey ellipsoid, anionic phospholipid; black ellipsoid, cholesterol; black squares, neutral lipid). With the exception of L-form bacteria and mycoplasma, the plasma membrane (PM) of most Gram-positive bacteria is covered by peptidoglycan (PGN), which is a major component of the bacterial cell wall. The cell wall of cocci and bacilli contain also lipoteichoic acid (LTA), whereas mycobacteria and corynebacteria contain arabinogalactan (AG) and outer membrane (OM) composed of mycolic acid, sugar and lipids.

Beyond the classification of bacteria according to Gram staining of peptidoglycan (PGN), bacteria can be subdivided by the cell shape into bacilli (rod-shaped) and cocci (sphere-shaped). Typical representatives of Gram-positive coccis form clusters (*Staphylococcus*), chains (*Streptococcus*) or tetrades (*Micrococcus*), whereas the Gram-positive bacilli can be thin, thick or branched. Bacteria with typical cocci form are rich in phosphatidylglycerol, whereas bacilli have in addition significant amounts of phosphatidylethanolamine (Table 2). A number of bacteria like actinomycetes and cell-wall deficient bacteria including mycoplasma and L-form bacteria show different morphologies that largely result from their differences in cell wall and plasma membrane lipid composition (Figure 2, Table 2). The molecular shape of actinomycetes looks to be somewhere in between rods and coccis organized in a branched network of hyphae, while mycoplasma and L-form bacteria can be round to oblong. The cells of cell-wall deficient bacteria are often observed as pleomorph and polymorph, but their colonies rather show egg-fried structures. Also, guanine and cytosine content of their genomes as well as their respiration type are often taken into consideration to differentiate between bacterial species or to explain their pathogenicity [49,50]. However, common to all bacteria is that the cytoplasmic membrane is surrounded by a cell wall. Those two compartments are separated by a periplasmic space or periplasm, which contains a wide variety of ions and proteins essential for numerous functions involving (electron) transport, substrate hydrolysis, degradation and detoxification. The cell wall of Gram-positive bacteria is made of many PGN layers of about 40–80 nm that stabilize the cell membrane [51]. In addition, the cell wall of Gram-positive bacteria contains a further unique component, teichoic acid that can be anchored to a glycolipid anchor with the plasma membrane or linked to the *N*-acetyl muramic acid unit of PGN. A cell wall does not exist in mycoplasma, L-form bacteria and some archaebacteria [52]. The plasma membrane is a phospholipid bilayer consisting of an inner and an outer leaflet (Figure 2), whereby the nature of phospholipids varies amongst the species not only in terms of their headgroups but also of their fatty acid moieties (Table 2). Basically, in comparison to Gram-negative bacteria, Gram-positive bacteria have a larger fraction of negatively charged phosphatidylglycerol (PG) containing saturated and unsaturated fatty acid with major distribution of C16:0, C18:0, C16:1 and C18:1, but also branched fatty acids like anteiso C15:0 and C17:0 chains.

### 3.1. Peptidoglycan, a Cell Wall Mesh

**Figure 3:**
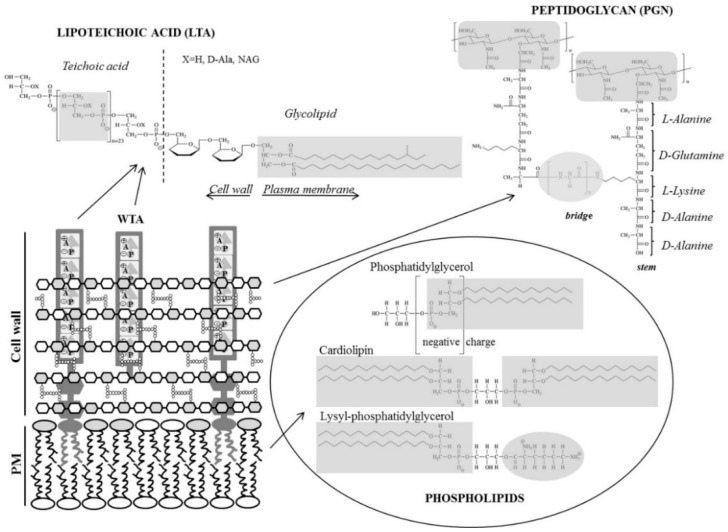
Schematic illustration of the cell envelope and main components of cell wall and plasma membrane (PM) of *S. aureus,* as a representative of Gram-positive bacteria.

Because of its rigidity, PGN determines the strength and characteristic cellular shape of bacteria, as shown e.g., for L-form bacteria from *Bacillus subtilis* [71] and in production of spheroplasts in *E. coli* [51,72]. PGN is a multi-gigadalton bag-like molecule and accounts for around 90% of the dry weight in Gram-positive and 10% in Gram-negative bacteria. The molecular weight of single layered *E. coli* PGN sacculus is 3 × 10^9^ Da, which is in the same range as a chromosome (2.32 × 10^9^ Da) of this bacteria [51]. In Gram-positive bacteria, the PGN network makes up 40–80 layers and is composed of alternating units of disaccharide *N*-acetyl glucosamine and *N*-acetyl muramic acid (NAG-NAM) cross-linked by a pentapeptide side chain (stem) [51,72] (Figure 3). The pentapeptide usually has the sequence l-alanyl-γ-d-glutamyl-diaminopimelyl-l-lysyl-d-alanyl-d-alanine [51,72]. In Gram-positive bacteria, an inter-bridge structure of five amino acid residues that varies between the species (e.g., five glycine molecules in *Staphylococcus aureus*) links two disaccharide-pentapeptide moieties [51,73]. PGN synthesis starts on the cytosolic side of the bacterial cell membrane from the common building block, lipid II (see e.g., [74]) that consists of a polyisoprenoid anchor of C_55_ carbons in a chain (11 subunits long, C_55_-PP) attached to one disaccharide-pentapeptide subunit via a pyrophosphate linkage. The lipid II monomer is translocated to the periplasmic (exterior) side of the bacterial cell membrane before it is incorporated into the growing PGN network. Different findings and models for organization of the PGN murein sacculus have been proposed and it has been a matter of debate if murein glycans and peptides are arranged parallel (layered model) or perpendicular (scaffold model) to the membrane [51,75,76]. Recent NMR studies revealed that the disaccharide backbone of Gram-positive bacteria adopts four-fold screw helical symmetry with disaccharide unit periodicity of 4 nm [73]. Each PGN stem is oriented 90° in respect to the previous stem and the lattice of crosslinked stems has parallel orientation.

### 3.2. Lipoteichoic Acid, an Anionic Polymer Matrix

Teichoic acids are important constituents of many Gram-positive bacteria, which are either tethered to the cytoplasmic membrane via a glycolipid (lipoteichoic acid, LTA) [77] or covalently anchored to the *N*-acetylmuramic acid of the peptidoglycan network (wall teichoic acid, WTA) [78] (Figure 3). Among other physiological functions, they play a role in determining the cell shape and in regulation of cell division [78,79]. For detailed information on LTAs, the reader is referred to a recent review by Reichmann and Gründling [80]. Wall teichoic acid polymers share a common linkage unit, but exhibit structural diversity in their repeat units. Four different classes of teichoic repeat units are described [79,81]. The basic structure of teichoic acid comprises a soluble polymer of glycerolphosphate or ribitolphosphate repeating units, which may vary from 15 to 50 residues between Gram-positive species. The glycolipid anchor in *S. aureus* is diglycosyl-1,2-diacylglycerol with two fatty acids of different composition [82], mostly C14:0 or branched C15:0 at the sn-2 position and C16:0, C18:0 or C20:0 at the sn-1 position of the glycerol moiety [83]. LTA deficient *ltaS* mutant of *S. aureus* exhibits aberrant cell growth and division. It is synthetic lethal with *tagO* mutant defective in WTA synthesis indicating that LTA and WTA compensate for their activities. Complete loss of the anionic polymer matrix in bacterial envelopes affects the growth leading to unviability of the cells [84].

In the outer membrane layer, the concentration of LTA ranges between 0.4% and 1.6% of the cell dry weight in the logarithmic growth phase [85], which means that the molecular ratio of phospholipid to LTA is around 10:1 [83,86]. In contrast to membrane lipids, LTA does not form a stable monolayer structure in aqueous dispersions [87], but a micellar supramolecular structure [88]. The structure of staphylococcal [88] and pneumococcal LTA micelles [89] was characterized by X-ray scattering revealing a total diameter of 22 nm. The core, made of hydrocarbon chains of the glycolipid anchor, is 5 nm, which is surrounded by an 8.5 nm shell of heavily hydrated hydrophilic chains. As observed for several bacterial species, the critical micelle concentration of LTA in phosphate-buffered saline ranges from 28 to 60 µg/mL [90]. Systematic thermodynamic studies on the miscibility of LTA with dipalmitoyl-phosphatidylglycerol (DPPG) revealed stable mixtures up to LTA concentrations of 20 mol % [87]. Incorporation of LTA in the DPPG matrix led to an increase of the phase transition temperature indicating a stabilizing effect on lipid membranes within the head group region [83]. At concentrations higher than 20 mol % phase separation occurs, accompanied by a destabilization of the lamellar aggregation of DPPG and segregation of LTA into the sub-phase, presumably in the form of micelles owing to the small cross-sectional parameter of the diacylglycerol moiety and the resulting conical shape of LTA [83].

### 3.3. Plasma Membrane Phospholipids

The main lipids found in Gram-positive membranes are summarized in Table 2. The phospholipid composition from one species to another may vary, but in general Gram-positive bacteria rather exhibit a high amount of phosphatidylglycerol (PG) and its derivates lysyl-phosphatidylglycerol (lysyl-PG) and cardiolipin (CL), respectively. To some extent, phosphatidylethanolamine (PE) is also present in Gram-positive cytoplasmic membranes. Moreover, phospholipids of Gram-positive species are characterized by a high content of branched-fatty acids, which were shown to influence the activity of AMPs [91]. The main physicochemical properties of these major bacterial phospholipids are summarized in Table 3. The major differences between PG and PE molecules, which are considerably important for the two-dimensional organization in membranes, relate to their net charge and their molecular shape. PGs are negatively charged at neutral pH, while PEs are zwitterionic. PEs in turn have an inverted cone-like molecular shape owing to the smaller headgroup area as compared to the cross sectional area required by the acyl chains and therefore are prone to form non-planar lipid aggregates, such as the inverted hexagonal phase, imposing a curvature strain onto the membrane [92,93]. Anionic lipids like PG with its charged headgroup occupy a larger area than predicted by its geometrical size owing to electrostatic repulsion resulting in looser packing [94]. The molecular shape of PG can be described as a cylinder, i.e., the cross sectional area of the headgroup matches the hydrophobic cross sectional area [95,96]. Moreover, PE lipids interact inter-molecularly by hydrogen bonding between the amino- and phosphate groups or carbonyl group of the sn2-chain, which favors closer lipid packing [97,98]. Therefore, PE will tend to adopt structures with higher intrinsic curvature, while PG will rather form flat lamellar lipid aggregates. Indeed, non-ideal mixing behavior and packing constraints were detected in binary lipid mixtures of di-saturated and mixed-chain PG/PE [99,100]. Thus, the molar ratio of PG and PE is important in determining lateral organization, packing and/or mobility, which can be amplified by the interaction with other membrane constituents, in particular by membrane-active molecules. For example, studies on the interaction of AMPs with DPPG/DPPE showed that both the human neutrophil peptide, HNP-2, and PGLa, a peptide from the skin secretion of the African clawed frog *Xenopus laevis*, discriminate between the different phospholipid subspecies inducing lipid segregation [31].

The physicochemical properties and phase behavior of CL are summarized in detail by Lewis et al. [102]. In brief, the relatively small size of the polar headgroup should promote greater cohesion between the CL hydrocarbon chains, which in turn should elevate the lipid phase transition temperature relative to those of most other phospholipids. In agreement, CL was shown to pack tightly, forming micro domains [101]. Further, the small size of the polar headgroup should also enhance the propensity of CL to form inverted non-lamellar lipid phases, which is attenuated by the presence of the mutually repulsive negative charges of the phosphate groups but promoted in the presence of divalent cations [103]. Nevertheless, the presence of CL in a membrane may still be a driving force towards an increase in membrane curvature strain. Haines et al. [101] described the head group as a bicyclic formation of the two phosphate groups linked to the central glycerol residue. Thereby, the H-bonding to the hydroxyl residue alters the pK_a2_ to >8 and creates, by trapping a proton, an acid-anion. Therefore, at physiological conditions, CL rather has a single negative net charge.

Numerous studies provide evidence that lipid composition determines the final morphology of every membrane and that the physicochemical properties of lipids, especially geometrical and physical constraints, contribute to a large extent to membrane organization. The first lipid domains were described for *E. coli* [104] and *B. subtilis* [105] found to be enriched in CL. They are located at the cell pole and septum. For these rod shaped bacteria, it was shown that the high cell-wall curvature at the poles and septum of bacteria is enriched in cardiolipin being essential for cell division [105,106,107]. Of note, as mentioned above, CL is a lipid prone to adopt non-bilayer structures of high curvature [103]. The shape of *E. coli* bacterium is suggested to be maintained by the separation of CL rich domains, as it is not well miscible with the major membrane constituent PE [108]. The same would be true for *B. subtilis,* which consists mainly of PG and lower amounts of PE and CL (Table 2). Notably, CL is also not very miscible with PG [109]. As well, PG was found to form distinct domains [110]. In *B. subtilis* it is distributed on helical structures along the cell surface, while in *E. coli* dot-like domains are observed. As illustrated recently, variability in the lipid composition of membrane domains may be a general factor shaping the interaction of membranes and proteins that is crucial for many different processes in bacteria including cell division [111,112]. 

Cell fractionation of *S. aureus* revealed that the cell wall makes up to 21% and membranes up to 10% of the cellular dry weight, whereby 90% of the lipids were found in the membrane and 10% in the cytoplasm [65]. In staphylococcal membranes, PG species make up to 95% of the total lipids [53]. No free fatty acids could be detected in *S. aureus* ([113]. Kuhn et al. [114] summarized a list of essential genes showing that mutations in *cdsA*, *pgsA* and *plsY* genes prevent synthesis of PG and its precursor acyl-glycerol-3-phosphate. Deletion of these genes is lethal. CL is the second most abundant lipid in Gram-positive bacteria, but generally it is present to a lesser extent in exponentially growing cells and rather accumulates in the stationary growing cells, where PG declines [115]. The same holds for lysyl-PG. Possible explanations to growth-related changes of phospholipid composition have been reported to include environmental conditions as well as regulation of cell cycle or DNA replication [114]. In addition, exposure to antibiotics that inhibit cell wall biosynthesis such as vancomycin, ampicillin or bacitracin stimulate the incorporation of lysine into PG [116], which seems to be also a protective mechanisms of Gram-positive bacteria against AMPs. This way, they reduce the overall negative charge on the surface by decorating the head group of PG with lysine, which with its two positive charges neutralizes the phosphate and confers a positive net charge to the modified lipid reducing the susceptibility to AMPs [117]. In fact, *mprF* mutants defective in synthesis of lysyl-PG showed increased susceptibility to a number of AMPs [114,118]. Information on chain packing and morphology of lysyl-DPPG was obtained in a differential scanning calorimetry and X-ray scattering study [119]. Under physiological conditions, the synthetic lysyl-DPPG resembled the parent lipid (DPPG) with respect to its melting behavior. However, in contrast to DPPG, lysyl-DPPG forms an interdigitated lamellar phase below the chain-melting transition owing to its large headgroup area, which will also affect the packing properties in bacterial plasma membranes.

It is interesting that actinomycetes e.g., corynebacteria, which usually have a shape between cocci and rods, the so-called coryneum, incorporate negatively charged phosphatidylinositol (PI) and its glycosylated derivatives in the membrane. This is needed to stabilize and strengthen their membranes, as they do not have glycosylated lipid anchors like LTA [120]. In addition to PGN, corynebacteria and mycobacteria have an outer membrane composed of trehalose derivatives, free lipid and mycolic acid, as well as a layer with arabino-galactan. All these components are missing in *Streptomyces*, another member of actinomycetes, which also display a strong variety of fatty acids. While in the cell wall of mycobacteria very long chain fatty acids C_60-90_ (α-alkyl, β-hydroxyl FA, mycolic acids) are found. In others, shorter carbon chains C_22-36_ consisting of a mixture of saturated and unsaturated FA occur [120]. The longer chain fatty acids are believed to play a role as a second permeability barrier functionally similar to the outer membrane of Gram-negative bacteria. Accordingly, mycobacteria contain both fatty acid synthase (FAS) I and II systems for de novo biosynthesis and elongation of fatty acids, whereas corynebacterium have only FAS I and *Streptomyces* has FAS II (for a review see [120]). *Streptomyces* and corynebacteria incorporate fatty acids not only in phospholipids but also in neutral lipids suggesting that the more cell wall components a bacterium loses, the more neutral lipids, e.g., triacylglycerol in *Mycobacterium tuberculosis* [62] or diacylglycerol in *Streptomyces hygroscopicus* [61], are found in their membranes. The same is true for mycoplasma, which are the smallest self-replicating prokaryotes having only a plasma membrane largely composed of PG or CL without cell wall or other lipid containing structures. However, they can uptake lipids from the environment or host and incorporate them in the membrane to stiffen them, e.g., by incorporation of sterol/sterol ester and triglycerides in *Mycoplasma homini*s [64] or PC and sphingomyelin in *Mycoplasma hyopneumoniae*, a pathogen isolated in China [121]. In accordance with this observation incorporation of cholesterol in cell wall-deficient *Acholeplasma laidlawii* abolished the gel-to-fluid phase transition [122,123]. Thus, maintaining membrane integrity makes the membrane of mycoplasma more similar to eukaryotic membranes and allows them to survive in a host. Normally, bacterial membranes do not contain sterol species.

Bacteria capable of mutating into a cell-wall-deficient “L-form” state proliferate by usual membrane deformation and scission process ranging from growth and shape distortion after losing the cell wall, to blubbing, tabulation and vesiculation [71]. Errington et al. [71,124] uncovered the crucial role of branched chain fatty acid synthesis in proliferation of the primitive cells of *B. subtilis*. Branched chain fatty acid deficient mutants went through pulsating shape changes, but failed to undergo membrane scission and separation of progeny, which was attributed to a reduction in membrane fluidity. To maintain an acceptable level of fluidity, bacteria alter their fatty acid composition by incorporation of lower melting fatty acids, such as unsaturated, short chain and branched chain fatty acids. In Gram-positive bacteria, modification of the ratio of saturated to unsaturated fatty acids and modification of the ratio of iso- and anteiso-fatty acids contributes to this adjustment. The importance of membrane composition for vesicle scission was demonstrated by optical microscopy experiments monitoring real-time incorporation of oleic acid into giant phospholipid vesicles explaining vesicle self-replication as a result of imbalance between membrane surface area and vesicle volume [125,126]. Thus, it was concluded that in order to proliferate, L-form bacteria compromise the loss of cell wall by intercalation of amphipathic molecules like fatty acids into the bilayer to increase the surface area above the internal volume of the cell that directly drives the shape distortion leading to vesicle fission.

## 4. Mode of Action of AMPs

The general view is that most AMPs act directly on cell membranes, but several in vivo studies have revealed that AMPs can interfere with a series of cellular processes and metabolic functions [24,127,128]. Intracellular targets such as nucleic acids and proteins have been proposed for those AMPs with very low membrane perturbing activity [129] via a “Trojan-horse” like mechanism for the peptide-uptake as shown e.g., for buforin II, which penetrates into *E. coli* cells and inhibits cellular function by binding to DNA and RNA [130] or cathelicidin-derived short Pro-rich peptides, which inhibit protein translation in 70S ribosomes or assembly of the large subunit of ribosomes [131]. A pioneering contribution to this aspect came from a study by Bob Hancock’s group [132]. They tested peptides of different structures including α-helical indolicidin variants differing in amphipathicity, charge, length and hydrophobicity, as well as a linearized bactenecin, Bac2A-NH_2_, against a panel of Gram-positive bacteria. All peptides exhibited membrane permeabilization, which however did not correlate with the antimicrobial activity of these peptides ranging from excellent to moderate. Although the exact mode of action of those peptides in Gram-positive bacteria has not been elucidated, based on the fact that at early time points membrane depolarization was not complete, even when more than 90% of bacteria had been killed, the authors concluded that cytoplasmic-membrane depolarization may not be the primary event for bacterial killing but that a certain level of permeabilization is required in order to reach an intracellular target. However, irrespective of the mode of action, AMPs have to interact first with the bacterial surface and to pass the cell envelope, before they can reach the cytoplasmic membrane or translocate into the cytosol. Thereby, electrostatic interactions between the cationic peptides and negatively charged components of the cell wall such as lipopolysaccharides (LPS) in Gram-negative and teichoic acids in Gram-positive bacteria will modulate the attraction to the bacterial surface [27]. In this respect, there is an ongoing debate to which extent interaction with cell wall components including peptidoglycan may entrap AMPs or promote accumulation of AMPs on the cytoplasmic membrane [45]. Besides considering this aspect, we will also discuss antimicrobial activity related to inhibition of the biosynthesis of cell wall components and mechanisms related to direct membrane damage.

### 4.1. AMPs Targeting Peptidoglycan

The PGN sacculus is often designated as a mesh, which is relatively porous and does not represent a permeability barrier for particles of approximately 2 nm and globular hydrophilic molecules of a maximum size of about 50 kDa that do not bind to PGN [133]. Note that most of the AMPs are between 15 and 50 amino acids, i.e., <6 kDa. Further, PGN is not negatively charged and hence is not considered to significantly entrap cationic AMPs, thereby reducing their effective concentration at the membrane surface. This assumption is in line with a recent study showing that PGN binding of a synthetic antimicrobial peptide, OP-145, developed from a screen of the human cathelicidin LL-37, did not affect its membrane permeability as tested in leakage experiments using PG liposomes in the presence and absence of 0.1 wt % PGN [27].

Two peptides that exhibit strong partitioning toward the PGN mesh are the human cationic polypeptide ECP (eosinophilic cationic protein) [134] and omiganan, a derivative of the bovine cathelicidin indolicidin, which has been in a phase III clinical trial [135]. ECP is an antimicrobial RNase taking part in the eosinophile-mediated inflammatory processes. It is around 155 amino acids long of which 19 are arginine residues, which results in a high isoelectric point of 11.4 and high cationicity [136]. These properties further confer high affinity to negatively charged surfaces. In model studies, however, ECP induced only weak leakage of negatively charged lipid vesicles composed of either POPG or POPG/POPC at its bactericidal concentration toward *S. aureus* [137,138]. Moreover, electron micrographs did not reveal any damage of the cell wall and no detectable lysis of *S. aureus* in the presence of ECP [134]. Similarly, although fluorescence quenching studies yielded high partitioning constants of omiganan for anionic model membranes, the peptide failed to induce leakage of those lipid vesicles. Thus, as omiganan incorporates into anionic bilayers without inducing severe membrane perturbations, it was suggested that the peptide translocates through the membrane and acts on an intracellular target such as DNA as reported for indolicidin [139]. The same may be the case for ECP. However, it is not clear if both ECP and omiganan actually have intracellular targets and if binding to PGN triggers accumulation of the peptides, enhancing the cytosolic uptake.

#### 4.1.1. Inhibition of PGN Biosynthesis

**Figure 4:**
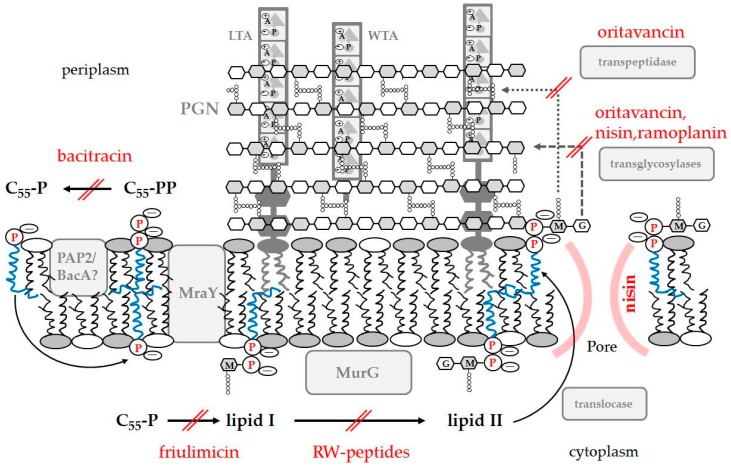
Proposed interaction sites of AMPs interfering with PGN biosynthesis. Nisin is typical of a number of lantibiotics and defensins that specifically bind to lipid II, though different binding motifs may apply. In addition, nisin can form a distinct peptide-lipid II pore indicated on the right hand side of the scheme. Short linear and cyclic RW-rich peptides are representative for peptides that delocalize peripheral membrane proteins like MurG, thereby interfering with physiological processes. Lipid II structure is simplified showing in addition to peptide and carbon chains the phosphate (P) with negative charges, *N*-acetylglucosoamine (G) and *N*-acetylmuramic acid (M).

In contrast to many antibiotics that exhibit their antimicrobial activity via binding to and inhibition of enzymes involved in PGN biosynthesis, AMPs bind to peptidoglycan precursors and in turn interfere with further enzymatic processes resulting in inhibition of PGN synthesis by sterically hindering the activity of enzymes [140]. Examples include the branched tricyclic glycopeptide vancomycin, which was developed in the 1950s and serves as a gold standard for the treatment of methicillin resistant *S. aureus* infections [141], as well as families of cyclic lipo(glyco)peptides and lipoglycodepsipeptides like the macrocyclic ramoplanin derived as a mixture of three components from *Actinoplanes* sp. [140,142]. Ramoplanin comprises a 49-membered macrocyclic depsipeptide substituted with a disaccharide and a lipid side-chain [143]. The peptide contains 17 amino acids of which several are of D-configuration and two positively charged ornithine residues, which in addition to the ring structure, are essential for antimicrobial activity [144]. Initially, it was suggested that ramoplanin binds to lipid I inhibiting MurG glycosyltransferase and hence block the biosynthesis of the key precursor lipid II [145]. As it seemed unlikely that ramoplanin readily translocates to the cytosol to have direct access to the intracellular lipid I, it was a matter of debate whether direct inhibition of the MurG catalyzed conversion of lipid I to lipid II can occur [74,142,146]. Later studies confirmed the higher affinity of ramoplanin toward lipid II forming a 2:1 stoichiometric complex thereby inhibiting the periplasmic transglycosylation step of peptidoglycan biosynthesis (Figure 4) [147]. Recently, it was reported that the peptide also induces membrane depolarization of methicillin-susceptible *S. aureus* at or above its minimal bactericidal concentration and induces dramatic morphological changes. These observations correlated with cell viability and thus it was suggested that this mechanism may also contribute to the antimicrobial activity of ramoplanin [148]. It seems to be a common pattern that AMPs interfering with PGN biosynthesis also have the potential to damage the cytoplasmic membrane, which would make them superior to other antimicrobial compounds having only one specific target.

Of all peptides that inhibit PGN biosynthesis, lantibiotics are studied in most detail. A comprehensive description of the mode of action of nisin-like class A lantibiotics as well as merscacidin-like class B lantibiotics is given in an excellent recent review [149]. In brief, nisin derived from *Lactococcus lactis* consisting of 34 amino acids inhibits primarily PGN synthesis but also disrupts bacterial membranes (Figure 4) [150]. In particular, the A/B-ring of nisin forms a cage with the pyrophosphate moiety of lipid II in a 1:1 stoichiometric complex, a common binding mechanism for these kinds of lantibiotics [151,152]. After initial binding, four of these complexes assemble with four additional nisin molecules to form a stable transmembrane pore. Using derivatives of lipid II with shorter aliphatic residues demonstrated that the lipid tail is not important for peptide binding, but is important for pore formation [153]. Nisin disrupted pure anionic phospholipid model membranes only at much higher concentrations than necessary for bacterial killing. However, in the presence of lipid II these liposomes become much more susceptible to nisin emphasizing the specific complex formation [154]. As supported by a number of studies [149], it is obvious that the membrane bound PGN precursor lipid II acts as a docking moiety to attract the nisin to the bacterial membrane and to promote peptide insertion into the membrane leading to permeation [155,156]. Mersacidins including plantaricin C have a different binding motif recognizing *N*-acetylglucoseamine and hence can discriminate between lipid I and lipid II inhibiting the transglycosylation step in PGN biosynthesis [157].

Oher peptides that share a similar mechanism of bacterial killing via lipid II targeting include defensins like human β-defensin 3, as well as the 11 amino acid depsipeptide teixobactin from the previous uncultured β-proteobacteria *Eleftheria therrae*, discussed in detail by Oppedijk et al. [149]. Targeting of lipid II was also observed by oyster defensins [158] and plectasin from the fungus *Pseudoplectania nigrella* [159]. Bacteria treated with oyster defensins accumulate the last soluble precursor of PGN, UDP-MurNAC pentapeptide without damaging the cytosolic membrane, suggesting that the peptides bind to lipid II after lipid II has been translocated to the outer leaflet of the cytoplasmic membrane [158]. Schmitt et al. [160,161] identified conserved residues involved in binding to lipid II of the structurally highly related plectasin and oyster defensins by comparing the sequences of these peptides. Phe2, Gly3, Cys4 and Cys25 were identified to be crucial for lipid II interaction and diversification at position 16 showed that the presence of positively charged residues improved the antimicrobial activity. The latter may be relevant for increased electrostatic interactions between defensins and the negatively charged membranes of bacteria [158]. This is consistent with their limited activity against Gram-negative bacteria, as lipid II is protected by the outer membrane preventing access of the oyster defensins to the periplasmic space and requires outer membrane damaging to become accessible in Gram-negative bacteria [158]. The same authors compared these with mammalian defensins, which have the same target lipid II. In contrast to oyster defensins mammalian defensins are active against both, Gram-negative and Gram-positive bacteria probably due to their membrane disruptive activity, which enables them to gain access to lipid II in Gram-negative bacteria.

Oritavancin, a semisynthetic lipoglycopeptide analogue of vancomycin, also displays a set of sequential mechanisms ranging from inhibition of PGN biosynthesis to perturbation of membrane integrity of Gram-positive organisms. Oritavancin binds to the alanine-alanine stem of the pentapeptide moiety of lipid II and also to the pentaglycyl bridging segment inhibiting PGN synthesis, thereby blocking transglycosylation and transpeptidation (Figure 4) [162]. In contrast to its analogue vancomycin, the 4´chlorobiphenyl group of oritavancin allows interaction with lipid II and cell membrane anchoring, which in addition results in perturbation of membrane integrity in *S. aureus* and *E. faecalis* [163]. Membrane depolarization in *S. aureus* following exposure to oritavancin was measured using the fluorescence indicator 3,3′-dipropylthiacarbocyanine [163,164]. Further, a “live and dead” assay, in which *S. aureus* living cells are stained with two fluorescent dyes, namely the membrane permeable Syto9 and membrane impermeable propidium iodide, showed that oritavancin treatment resulted in displacement of Syto9 by propidium iodide, which clearly indicates damage of the cell membrane resulting in increased permeability [164]. As well in model systems, oritavancin induced rapid leakage of liposomes composed of lipids extracted from *S. aureus* [165], CL/POPE and POPG/POPE [166]. It is tempting to speculate to which extent the ability of oritavancin to interact with phospholipids, to permeabilize those lipid vesicles at bactericidal concentration and to inhibit PGN biosynthesis contributes to its activity. In any case, such multiple facets of molecular mechanism are favorable to overcoming bacterial resistance. Thus, high-level oritavancin resistance has not yet been reported, neither in the laboratory nor in clinical studies [162,167].

Bacitracin, a common antibiotic used for the treatment of bacterial infections caused by Gram-positive bacteria, targets the C_55_-isoprenyl (undecaprenyl) pyrophosphate carrier (C_55_-PP), another essential molecule in PGN biosynthesis. This antibiotic is a mixture consisting of cyclic polypeptides produced by *Bacillus subtilis* and *Bacillus licheniformis*. In contrast to most AMPs, the overall charge of bacitracin is neutral. The commercial products contain 70% bacitracin A, a dodecapeptide of 1.4 kDa, which is the most potent, as well as an amphipathic one within the mixture that undergoes complexation with C_55_-PP in the presence of divalent metal ions [168]. The consequence is inhibition of de-phosphorylation of this carrier catalyzed by PAP2 (type 2 phosphatidic acid phosphatase) and BacA (C_55_-PP phosphatase) and results in the inability of the cell to transport this carrier from the outer to the inner membrane leaflet necessary for the cell wall synthesis in the growing cell (Figure 4).

Finally, friulimicin is an antimicrobial lipopeptide that also requires divalent metal ions to build an antibiotic-metal-lipid complex. Also amphiphilic, friulimicin is produced by the actinomycete *Actinoplanes friuliensis* and exhibits excellent activity against Gram-positive pathogens. It consists of a macrocyclic decapeptide core and a lipid tail, interlinked by an exocyclic amino acid, and possesses an overall negative charge with amphipathicity increasing in the presence of calcium [169]. This peptide is supposed to form a complex with the cytosolic lipid carrier C_55_-P in the presence of calcium and to inhibit its conversion to lipid I mediated by Mra Y transferase (Figure 4) [169]. Friulimicin shares structural properties with the lipopeptide daptomycin from *Streptomyces roseosporus*, which is also negatively charged and selectively kills Gram-positive bacteria [170]. Although several membrane damaging mechanisms are proposed for the mode of action of daptomycin, it has not been excluded that aggregation of daptomycin in the membrane would interfere with membrane-associated processes including e.g., cell division (see Section 4.3).

#### 4.1.2. Binding to PGN: Recognition and Elimination of Pathogens

Bacteria are detected by the innate immune system through a series of pattern recognition receptors. Peptidoglycan recognition proteins such as lectins and natural or semi-synthetic antibiotics like glycopeptides bearing unusual amino acids or modifications belong to pattern recognition molecules that target this unique cell wall component [140,171,172]. These evolutionary highly conserved proteins directly attack pathogens and induce an innate immune response, whereby binding to PGN initiates bacterial killing. Typical representatives of such proteins are ~16 kDa C-type lectins, which possess a characteristic globular structure with four functional domains. This functional region encompasses a carbohydrate binding domain, a neck repeat region of tandem helical repeats with exposed hydrophobic residues allowing oligomerization and a transmembrane domain as well as a cytosolic domain promoting internalization into the membrane [173]. Many of these proteins bind to PGN in a Ca-dependent manner, while human RegIIIα (also known as HIP/PAP, hepatointestinal pancreatic/pancreatitis associated protein) recognizes the PGN backbone in a Ca-independent manner [172]. NMR studies showed that the Glu-Pro-Asn tripeptide motif located in the long loop region of human C-type lectins is essential for binding to PGN carbohydrate [172]. In RegIIIα, an exchange of the Glu residue in this motif by Gln prevents binding to PGN resulting in a reduced affinity to *staphylococcal* PGN and decreased antimicrobial activity against Gram-positive *Listeria monocytogenes*. Although the mechanism of bacterial killing by lectins is not fully understood, a recent publication demonstrated that RegIIIα acts via formation of a membrane-penetrating pore [174]. While binding to PGN is crucial for attachment to bacteria, it is not necessary for subsequent pore formation, as shown for RegIIIα that permeabilized membranes of non-spore forming *Listeria monocytogenes* and anionic phospholipid vesicles by forming a hexameric pore with a diameter of about 100 Å [174]. Uncontrolled ion efflux and subsequent osmotic lysis resulted in bacterial killing. Interestingly, RegIIIα has no activity toward Gram-negative bacteria, as LPS inhibits RegIIIα membrane permeabilization and disruption of liposomes composed of *E. coli* total lipid extracts [174].

Although proteins such as C-type lectins are bigger in size and more complex in structure than the small AMPs, they still share basic functions like membrane disruption to kill pathogens. Similarly, the complement system of plasma proteins that are part of classical, alternative and lectin pathway acts as a rapid and efficient immune surveillance system by forming a membrane attack complex that results in direct osmotic lysis of cells [175,176]. Activation of one or another complement pathway is dependent on the membrane composition of the pathogen of which specific targets like charged patterns, array of repeating sugar residues, LPS, etc. are recognized and neutralized [176]. Thus, during evolution, nature has developed versatile strategies to recognize and eliminate pathogens.

### 4.2. Teichoic Acid: Enhancing or Blocking AMP Activity

Many AMPs that exhibit membrane disrupting properties like LL-37 [39,177], melittin and cecropin [178], also efficiently bind to teichoic acids [45,179]. Hence, there is an ongoing debate to which extent binding to wall or LTA hinders or promotes the interaction of AMPs with the cytoplasmic membrane [27,45]. Considering that the structure of LTA (e.g., from *S. aureus)* contains on average 24 glycerolphosphate repeating units of which each contains one negative charge from the phosphate group, it is obvious that LTA can potentially attract positively charged AMPs [179,180,181]. Therefore, it is not unlikely that AMPs may be entrapped by teichoic acids through increased peptide adsorption to these cell wall components, resulting in a decrease in local peptide concentration on the cytoplasmic membrane. In this context, it is of interest that it was shown that bacterial killing via membrane disruption only occurs when the membrane surface is completely saturated with AMPs [28]. Thus, trapping of peptides by LTA may sufficiently reduce the total concentration of peptides on the cytoplasmic membrane, preventing full membrane coverage and in turn membrane disruption. Reduction of the effective peptide concentration on the cytoplasmic membrane due to the presence of LTA can be deduced from studies on membrane mimetic systems with OP-145, a derivative of the human cathelicidin LL-37 [182]. Incorporation of LTA into POPG liposomes decreased the rate of leakage at a given lipid-to-peptide molar ratio, however, at very high peptide concentration full leakage was also achieved. The membrane permeability observed for those model systems was in the range of the effective lethal concentrations for *S. aureus* at 1.6–3.2 µM. Therefore, it was proposed that the molecular mechanism of bacterial killing by OP-145 can still be explained by membrane perturbation [182]. Likewise, isolated LTA decreased the antimicrobial activity of the membrane damaging peptides LL-37 and mellitin [179]. A specific case is the antibacterial 7 kDa polypeptide B chain of ß-bungarotoxin, the main presynaptic phospholipase A_2_ neurotoxin from snake venom. Membrane damaging activity owing to its abundant positively charged amino acid residues was shown by calcein release from liposomes composed of binary mixtures of PG/PE and PG/CL, respectively [183]. However, when the peptide was pre-incubated with LTA, calcein release from PG/CL vesicles was completely abolished in agreement with the finding that the peptide was unable to inhibit growth or induce membrane permeability of *S. aureus.* Upon binding to LTA, circular dichroism spectra indicated that the peptide undergoes conformational changes resulting in inhibition of its active site that abrogates its membrane-damaging activity and inhibits its bactericidal activity toward *S. aureus* [184].

Another view of the same problem is that binding of AMPs to teichoic acids may initiate bacterial killing by mediating the entry of peptides towards the cytoplasmic membrane. In other words, by building a poly-anionic ladder, LTA and WTA may help poly-cationic peptides to traverse from the outside to the cytoplasmic membrane. Thereby, WTA extends beyond the PGN network, while LTA may not be able to reach past this layer [78]. Accordingly, Koprivnjak et al. [185] suggested that the killing activity of both the 14 kDa mammalian group II phospholipase A2 (gIIA PLA_2_) and the highly basic 45 amino acid human β-defensin 3 toward *S. aureus* depends on the initial electrostatic interaction with WTA, as the *tagO* mutant lacking WTA was selectively resistant to these AMPs. In contrast, negligible differences were observed in the lipase activity and binding of gIIA PLA_2_ toward cell-wall depleted protoplasts from both wild type and *tagO* mutant of *S. aureus* suggesting that binding of gIIA PLA_2_ to WTA is important to facilitate cell wall penetration and to gain access to the cytoplasmic membrane.

The impact of binding to teichoic acids on the antimicrobial activity of AMPs can also be deduced from a common resistance mechanism of Gram-positive bacteria, which is D-alanylation of the LTA glycerolphosphates [82,186] to reduce the overall negative charge on the surface, lowering the affinity to cationic molecules like AMPs. Indeed, reduction of the D-alanyl content of the cell wall increases the susceptibility to AMPs [187,188,189,190]. Accordingly, depletion of D-alanylester from teichoic acid in *dlt* mutants from gallidermin-sensitive *S. aureus* resulted in increased susceptibility to positively charged AMPs such as defensins, protegrins, magainin and diverse lantibiotics but not to neutral gramicidin B. This finding indicates the importance of electrostatic interactions between *S. aureus* and AMPs [189]. In group B Streptococci, however, D-alanylation showed little influence on the surface-association of fluorescently-labeled AMPs [191], which suggests that charge effects are not solely responsible for the observed changes in susceptibility to AMPs. Here, the protective effect was explained by the increased cell wall density observed in strains with D-alanylation preventing penetration of cationic AMPs.

#### 4.2.1. Direct Killing by Binding to LTA

Finally, another bactericidal protein, the phospholipoglycoprotein vitellogenin, which is a major precursor of the yolk proteins in oviparous organisms, kills bacteria via binding to LTA and not via membrane disruption [181]. Results from scanning electron microscopy demonstrated that 450 kDa vitellogenin from the fish *Hexagrammos otakii* causes damage of the cell wall of *S. aureus* whole cells with the appearance of collapsed architecture, but does not alter the morphology of cell wall deficient *S. aureus* protoplasts. Severe cell lysis by vitellogenin was only observed for *S. aureus* whole cells, but not in cell wall depleted protoplasts. In addition, destroying of *S. aureus* cell-wall is abolished, when vitellogenin was pre-incubated with LTA before applying to *S. aureus*, which concomitantly resulted in loss of antibacterial activity of the peptide. These observations suggest that the binding of vitellogenin to LTA is lethal to *S. aureus* rather than attacking the plasma membranes [181]. In addition, vitellogenin may act as a multivalent pattern recognition molecule capable of binding to LTA, lipopolysaccharide, glucan and virons [192], as also suggested for vitellogenin from scallops [193].

### 4.3. Interaction of AMPs with the Cytoplasmic Membrane

As mentioned earlier, a large number of AMPs supposedly target the cytoplasmic membrane leading to membrane disruption and/or disorder and consequently to bacterial death. So far, there is consensus that the very distinct lipid composition of bacterial and mammalian cytoplasmic membranes mainly manifested as differences in surface charge and bilayer fluidity favors binding of cationic AMPs to the anionic bacterial membrane, thus allowing AMPs to discriminate between bacteria and host cells [30,47]. In addition, hydrophobic effects have to be considered not only for membrane partitioning but also for binding [194]. Thus, the physicochemical properties of AMPs especially their amphipathicity determine their membrane activity [46]. In the majority of cases, the mechanisms of membrane perturbation at a molecular level are not yet clear, although numerous models exist to explain the membrane damaging activities of AMPs [195]. In general, however, the mode of action of AMPs is mostly discussed within the framework of the carpet [196] or pore model [197].

One example is alamethicin, a 20 residue amphipathic α-helical peptide derived from *Trichoderma viride*, which contains non-proteinogenic amino acid residues and which due to the glutamic acid at position 18 has a net negative charge at pH 7 [198]. Its high activity against Gram-positive bacteria but negligible activity against Gram-negative bacteria was attributed to the hydrophobicity of the peptide, which may prevent translocation of the peptide across the outer membrane to reach the cytoplasmic membrane of Gram-negative bacteria [48]. The high bactericidal activity of alamethicin against mollicutes, which do not have complex cell wall architecture, would be in line with this argument [48]. On a molecular level, a barrel-stave type pore was proposed from neutron and X-ray analysis of oriented membrane model systems [199] and recently directly visualized using high resolution electrochemical scanning tunneling microscopy [200]. At low concentrations, alamethicin aligns parallel at the membrane surface, but at a certain threshold concentration inserts perpendicular into the lipid bilayer forming a transmembrane pore. This barrel-stave type pore consists of eight monomers, where the hydrophobic face of alamethicin is oriented toward the hydrophobic core of the membrane and the hydrophilic face toward the pore lumen [201]. Huang’s group investigated a number of naturally occurring peptides including magainins, melittin and protegrins, but only found the barrel-stave type pore for alamethicin [202]. The other peptides appear to induce a toroidal pore, in which AMPs together with the lipid form a water-filled pore in a way that the lipid monolayer bends continuously through the pore so that the water core is lined by both the peptides and the lipid headgroups [197,203]. It should be noted however that these experiments were performed using PC and PC/PG, respectively, which may not represent the most appropriate bacterial mimetic system.

Apart from membrane disruption by formation of a pore complex between AMPs and lipid II as described above, there is no membrane-disrupting mechanism that could be considered to be typical for Gram-positive bacteria. The molecular mode of action discussed for AMPs to disrupt membranes such as the carpet model [196], detergent-like [204] or interfacial activity [205] can be relevant for both, Gram-positive and Gram-negative bacteria. Therefore, we focus our discussion on experiments that are directly related to membrane effects in Gram-positive bacteria and on those aspects that might make a certain molecular mechanism more likely to occur in Gram-positive bacteria.

#### 4.3.1. Lipid Segregation and Alteration of Membrane Domains as a Mode of Action of AMPs

Interaction of AMPs with specific membrane domains, which are involved in bacterial cell differentiation, cell division and protein secretion, has been also reported for cationic AMPs. For example, in *E. faecalis* at sub-inhibitory concentrations human β-defensin 2 disrupted the localization of SecA ATP translocase and sortase SrtA, which play a role in the attachment of virulence factors occurring at spatially restricted domains containing anionic lipids [206]. Similar effects were found upon incubation of *S. pyogenes* with sub-inhibitory concentrations of the human neutrophil peptide 1 and polymyxin B [207]. These observations suggest that disruption of the assembly of virulence factor may be an additional mode of action of AMPs. Further evidence that cationic AMPs target membrane domains enriched in anionic lipids comes from studies both on model systems and bacterial cells. For instance, cecropin A [208] as well as *N*-acyl derivatives from lactoferricin [209] remodeled PE/CL domains present at the septum and poles of *E. coli* resulting in cell division defects. Preferred partitioning into PE/CL mixtures was also demonstrated for cyclic RW-rich hexapeptides, which was governed rather by hydrophobic than electrostatic effects [210,211]. Again, the results from the membrane mimics were in agreement with fluorescence staining experiments of *B. subtilis* membranes, which revealed that the short peptide interfered with the CL-enriched domain organization at septum and pole [211]. The central role of these negative intrinsic curvature lipids for cell division is to provide the proper lipid packing constraints at the division site and the cell poles [107,212,213]. Furthermore, localization of proteins that regulate the division plane such as MinD and MinE to *E. coli* membrane is CL and PG dependent [214]. Thus, it was proposed that anchoring of those proteins and other peripheral membrane proteins might be disturbed owing to the altered domain organization by the hexapeptide [211]. This assumption is supported by studies on linear RW-rich hexapeptides, which demonstrated that these peptides dislocate peripheral membrane proteins such as MurG (Figure 4) and cytochrome c in membranes of *B. subtilis* interfering with cell wall biosynthesis and energy metabolism, respectively [215,216]. Again, this was attributed to preferential interaction with PG leading to a change in membrane domain organization. The same phenomena were observed with the cyclic peptide gramicidin S. Therefore, specific interaction of AMPs with anionic lipids will have important implications for the structure and integrity of membranes and hence influence the function of membrane proteins adversely affecting viability of bacteria.

In model systems, lateral phase separation in bilayers composed of anionic and zwitterionic lipids has been demonstrated for a number of cationic AMPs because of their preferential binding to the negatively charged lipid components [31,217,218,219,220,221]. Although phase separation was also observed for mono-component systems, i.e., formation of peptide-poor and peptide-rich domains [32,182], promotion of lateral phase separation has been mainly investigated using PE/PG and PE/CL mixtures. Laterally, heterogeneous domains with a preferred location of AMPs in one domain will differ significantly in their properties with respect to membrane thickness, fluidity and curvature strain. Consequently, at the boundary of these domains, packing defects will occur, which will lower the membrane permeability barrier representing a general mode of action of AMPs, in addition to interfering with membrane structure and organization [29,30,44]. Lateral phase separation has been specifically proposed as a mechanism contributing to the antimicrobial activity of a designed α/β peptide [222] and an oligo-acyl-lysine (OAKs) compound [223,224]. Further studies indicated that lipid segregation is not primarily dependent on the nature of the anionic headgroup and acyl chain [223]. This correlates with findings that the inhibitory activity of short OAK peptides against Gram-positive bacteria was dependent on the fraction of anionic phospholipids in the cytoplasmic membrane, i.e., it was high against staphylococci, streptococci and enterococci but low for listeria and bacilli [225]. Notably, a study on the interaction of different cyclic hexapeptides with PE/PG lipid membranes showed that in addition to electrostatic binding, distinct positioning of the three hydrophobic amino acids also contributed to binding affinity and in particular to the extent of induction of lipid phase separation, which correlated with antimicrobial activity [210]. Clustering of anionic lipids was shown to be most effective for substances with sequential positive charges contained within a flexible molecule that can adapt to the distances of charged groups on the surface of the bacterial cell membrane [226]. This implies for the design of AMPs that peptides with sequences rich in cationic residues and conformational flexibility will be most prone to act via such a mechanism [221,226].

#### 4.3.2. A Role Model for AMPs: The Multifaceted Actions of Daptomycin 

**Figure 5:**
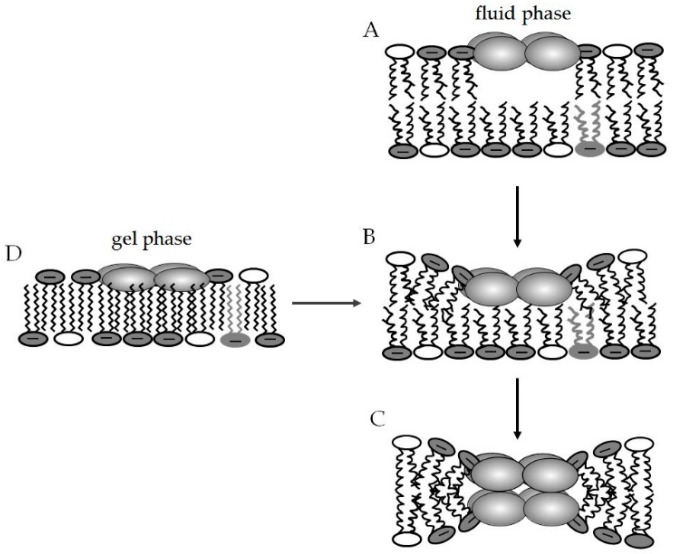
Scheme of proposed pore formation of daptomycin modified from [231]. Insertion of the peptide in the membrane interface and oligomerization creates a void in the hydrophobic core (**A**) inducing local positive curvature accompanied by deeper penetration of the peptide complex (**B**). This results in a dimple formation with a locally strongly decreased membrane thickness [236]. The oligomeric daptomycin may flip to the inner membrane leaflet and eventually combine with a complex of the outer leaflet to form a toroidal pore (**C**). Note, in the gel phase the void can be compensated by interdigitation of the lipid’s acyl chains (**D**) as shown for several AMPs [234].

Pore formation, though less defined, was also suggested for the cyclic lipopeptide daptomycin from *Streptomyces roseosporus*, which selectively kills Gram-positive bacteria including methicillin-resistant *S. aureus* and vancomycin-resistant *Enterococcus* [170]. In the presence of calcium daptomycin, which at neutral pH has a net negative charge of three, forms micellar aggregates in aqueous solutions. Upon interaction with membranes, these micelles may need to dissociate before the peptides insert in the outer leaflet of the bilayer. This process is facilitated by calcium, which strongly binds to negatively charged lipid headgroups. It was suggested that insertion of daptomycin may be accompanied again by oligomerization inducing a positive curvature strain on the membrane phospholipids to form a pore and in turn leakage of potassium from the bacterial cell leading to loss of membrane potential, dysfunction of macromolecular synthesis and finally to cell death [170,227,228,229]. In general, promotion of positive curvature strain was suggested to play a role in toroidal pore formation, but may be also involved in micellization of a bilayer [230]. However, when CL was added at molar fractions of 10–20 mol % to PG liposomes, pore formation was prevented [231]. This finding was explained by different penetration depths of daptomycin in PG and PG/CL mixtures (Figure 5). Thereby, the extra bulk in the acyl chain layer of CL should enable deeper penetration of the oligomeric daptomycin complex avoiding the creation of voids in the hydrophobic core. Voids are energetically unfavorable and can be compensated by increased trans-gauche isomerization of the acyl chains (increase of disorder), moving of the inner bilayer leaflet towards the outer (interdigitation) or bending of the leaflets (curvature induction) [29,44,232,233]. If latter is the case, as proposed for the system daptomycin/PG, positive curvature induction would result in the formation of a half-toroidal structure [231]. The authors argue further that this should enable the oligomeric peptide complex to flip to the inner leaflet and then combine with a second complex in the outer leaflet to form the final pore (Figure 5). Thus, it may be also conceivable that CL, which in particular in the presence of calcium adopts lipid structures of negative curvature [103], counteracts the positive curvature needed for such a pore formation. In fact, such a model includes important phenomena such as induction of curvature strain and membrane thinning owing to increased acyl chain disorder or interdigitation as observed for a number of AMPs. For example, α-helical peptides that align parallel to the membrane surface such as the bee venom toxin melittin, the frog skin antimicrobial peptide PGLa, human cathelicidin LL-37 and its derivative OP-145 are prone to induce interdigitation [182,234]. The impact of void formation and thus bilayer destabilization was demonstrated by varying both the nature of headgroup to modulate the depth of peptide penetration (less for anionic lipids) and acyl chain length to modulate the possible volume of void formation (the longer the chain the larger the void) [234]. This kind of membrane perturbation may be more relevant for Gram-positive bacteria because of the larger fraction of anionic membrane lipids. Induction of curvature strain that even led to gross morphological changes, i.e., formation of cubic lipid aggregates, was reported for lipid extracts from *A. laidlawii* in the presence of the cyclic decapeptide gramicidin S [230,235].

A completely new view on the mode of action of daptomycin was presented in a very recent study [237]. Using giant unilamellar vesicles, evidence was provided that above the threshold concentration the peptide exerts a lipid extracting effect. Like in the other studies, this phenomenon was only observed when the vesicles contained PG and when calcium was present. Moreover, the extra-membranous aggregates appeared to be similar to the direct action of daptomycin on bacterial membranes [238]. Interestingly, similar observations were reported from amphotericin B that presumably kills yeast by extracting ergosterol from lipid bilayers [239]. It was suggested that such a mechanism may also apply to other membrane-active compounds such as AMPs [240]. Chen et al. [237] stressed, however, that the lipid extracting effect of daptomycin was inadequate to explain effects related to CL, which indicates that daptomycin may have various membrane targets.

This assumption is strongly supported by the kind of mutations that confer resistance to daptomycin, which were found to be related to lipid synthesis, e.g., mutants being defective in synthesizing lysyl-PG (*mprF*) in *S. aureus* [241], cardiolipin-synthase (*cls*) in *E. faecalis* [242] or phosphatidylglycerol-synthase (*pgsA*) in *B. subtillis* [243]. In fact, it was shown that binding of daptomycin to PG-rich membrane domains in *B. subtilis* results in delocalization of the cell division protein DivIVA [238]. As a consequence, dramatic cell wall and membrane defects lead to anomalous cell morphology and eventually to membrane disruption. Further, it is known that DivIVA preferentially locates in membrane regions of high curvature [244] and hence it was assumed that membrane domains formed by daptomycin are characterized by such a curvature. This may also explain the strong association of daptomycin with the bacterial division septum by its preferential insertion into the leading edges of highly curved septal and forespore membranes [238]. Furthermore, in *E. faecalis*, daptomycin also interacts with the division septum, which correlates with the presence of CL domains [245]. Altered targeting was found in daptomycin-resistant *E. faecalis*, where a point mutation in LiaF that senses AMPs and regulates CL septal localization, leads to redistribution of CL away from the division septum, thus preventing daptomycin from binding to this region.

In summary, although the exact molecular mechanisms of action of daptomycin are still disputed, this clearly exemplifies that AMPs may interfere with membrane structure and membrane function at various levels by different means. Thus, AMPs may induce (i) membrane permeabilization; (ii) disrupt membrane domain organization and (iii) delocalize peripheral membrane proteins, which may make such peptides superior to antibiotics that act only on one specific target.

## Figures and Tables

**Table 1:** Characteristic physicochemical parameters of AMPs.

	Antimicrobial Activity		
	Anti Gram-Positive	Anti Gram-Negative	Broad Spectrum
peptide length			
<10 AA	16	5	63
10–50 AA	361	153	1890
50–100 AA	49	37	211
100–150 AA	6	10	35
net charge			
>20	0	0	11
11–20	3	4	57
6–10	51	28	415
1–5	308	145	1212
0	44	14	112
<0	26	17	100
% hydrophobic residues			
<10	1	6	23
11–20	3	4	57
21–30	27	26	155
31–40	136	41	544
41–50	103	73	704
51–60	76	26	440
61–70	76	10	264
71–80	3	1	20
81–90	1	1	3
>90	1	0	3
secondary structure			
unknown	305	127	1385
helix	38	19	348
beta-strand	8	7	77
helix & beta strand (unpacked)	4	0	3
helix & beta strand (packed)	16	3	59
disulfide bonds	52	23	236
rich in unusual AA	0	23	70
Total AMP entries ^1^	432	205	2199

^1^ number of AMPs found in the Antimicrobial Peptide Database in each linkage [16].

**Table 2:** Major lipid species detected in cytoplasmic membranes of representative Gram-positive bacteria.

Organism	Morphology Cell/Colony	Weight Percentage of Total Lipid of Cell Membrane							Major Fatty Acids	References
		PG	lysyl-PG	PI ^a^	PE	CL	NL	Others ^b^		
Cocci	round/chain									
*Staphylococcus aureus*	round/cluster	43	30	-	-	22	-	5	ai-C15:0; C18:0 ai-C17:0; C20:0	[53,54]
*Enterococcus faecium*	round/diplococci	33.8	14.4	-	-	39	-	12.8	C16:0; C16:1w7c C18:1w7c	[55]
*Streptococcus sanguis*	round/diplococci	82	-	-	-	18	-	-	C18:1; C16:1; C16:0	[56,57]
*Streptococcus pneumoniae*	round/cocci lancet shape	50	-	-	-	50	-	-	C16:0; C16:1; C18:0	[58]
Actinomycetes	spherical pleomorph/filamentous									
*Corynebacterium glutamicum*	rods/V-shaped	72.4	-	13.1	-	14.1	-	0,4	C16:0; C18:1	[59,60]
*Streptomyces hygroscopicus*	rods, mycelium	-	-	7	36	16 ^c^	30	11	i-C16:0; i-C14:0; i-C15:0	[61]
*Mycobacterium tuberculosis*	long, slender rods/filamentous	-	-	13	5	6	54	22	C16:1, C18:Me	[62,63]
Cell wall deficient	polymorph/fried egg									
*Mycoplasma hominis* ^d^	round to oblong/fried egg	33	-	-	-	-	60	7	C16:0; C18:0; C18:1	[64]
L-form *S. aureus*	polymorph/fried-egg	26	17	-	-	54		3	ai-C15:0; C18:0 ai-C17:0; C20:0	[53,65]
L-form *S. hygroscopicus*	polymorph/fried-egg	-	-	13	37	22	16	12	ai-C15:0; ai-C17:0, i-C16:0; C18:2 ^e^	[61]
Bacilli	round-ended cylinders/single or in chains									
*Clostridium difficile*	large, blunt-ended rods/pairs or chains	100	-	-	-	-	-	-	C16:0; C16:1 C18:0; C18:1	[66]
*Bacillus subtilis*	Rods/chain	70	-	-	12	4	-	14	ai-C15:0; i-C17:0	[67,68,69]
*Listeria monocytogenes*	slender, short rods/single or in chains	29	23	-	9	22	-	17	ai-C15:0; i-C15:0; i-C17:0	[70]

^a^ PI+PI mannosides; ^b^ “Others” corresponds mainly to glycolipids besides of traces of other lipids; ^c^ CL+CL derivatives; ^d^ sterol-requiring mycoplasma; ^e^ major fatty acids only in neutral lipids (NL); i, iso-branched; ai, anteiso branched fatty acids.

**Table 3:** Typical physicochemical properties of Gram-positive bacterial phospholipids.

	PG	lysyl-PG	CL	PE
Net charge	−1	+1	−2 (−1) *	0
H-bonding ability	no	no	yes	yes
Molecular shape	cylindrical	truncated cone	inverted truncated cone	inverted truncated cone
Intrinsic curvature	zero	positive	negative	negative
Organization	bilayer	bilayer	inverse micelles	inverse micelles

* for details see text and [101].

## Abbreviations

The following abbreviations are used in this manuscript:

AMPs	antimicrobial peptides
CL	cardiolipin
DPPE	1,2-dipamitoyl-sn-glycero-3-phospho-rac-ethanolamine
DPPG	1,2-dipamitoyl-sn-glycero-3-phospho-rac-glycerol
LPS	Lipopolysaccharide
LTA	lipoteichoic acid
LUV	large unilamellar vesicle
lysyl-PG	lysyl-phosphatidylglycerol
MLVs	multilamellar vesicles
NAM	N-acetyl glucosamine
NAG	*N*-acetyl muramic acid
PA	phosphatidic acid
PC	phosphatidylcholine
PE	phosphatidylethanolamine
PGN	peptidoglycan
POPC	1-palmitoyl-2-oleoyl-sn-glycero-3-phosphocholine
POPE	1-palmitoyl-2-oleoyl-sn-glycero-3-phospho-rac-ethanolamine
POPG	1-palmitoyl-2-oleoyl-sn-glycero-3-phospho-rac-glycerol
PS	phosphatidylserine
PI	phosphatidylinositol
WTA	wall teichoic acid
